# Comparative analysis of host resistance to *Sarcoptes scabiei* var. *cuniculi* in two different rabbit breeds

**DOI:** 10.1186/s13071-019-3764-5

**Published:** 2019-11-08

**Authors:** Wenrui Wei, Yongjun Ren, Nengxing Shen, Hongyu Song, Jing Xu, Ruiqi Hua, Haojie Zhang, Christiana Angel, Xiaobin Gu, Liangde Kuang, Yue Xie, Xuerong Peng, Xiaohong Xie, Guangyou Yang

**Affiliations:** 1 Department of Parasitology, College of Veterinary Medicine, Sichuan Agricultural University, Wenjiang, 611130 China; 2 Sichuan Animal Sciences Academy, Chengdu, 610066 Sichuan China; 3 Animal Breeding and Genetics Key Laboratory of Sichuan Province, Chengdu, 610066 Sichuan China; 4 Department of Chemistry, College of Life and Basic Science, Sichuan Agricultural University, Wenjiang, 611130 China; 5 Department of Veterinary Parasitology, Faculty of Veterinary Sciences, Shaheed Benazir Bhutto University of Veterinary and Animal Sciences, Sakrand, 67210 Sindh Pakistan

**Keywords:** *Sarcoptes scabiei*, Rabbit breeds, Host resistance, Antibodies levels, *MHC-DQA*

## Abstract

**Background:**

Scabies, caused by infestation of the mite *Sarcoptes scabiei*, is one of the most severe ectoparasitic diseases in rabbits. Scabies seriously affects the commercial rabbit breeding, causing severe economic losses. Host resistance to *S. scabiei* is an important factor in further development of the rabbit industry. In the present study, we compared the host resistance to *S. scabiei* var. *cuniculi* of a new breed of domestic rabbit propagated by the Sichuan Animal Sciences Academy (QiXing rabbit, QX) compared with that of a traditional rabbit breed in the domestic rabbit industry (IRA rabbit, IRA).

**Methods:**

Both QX and IRA rabbits were experimentally infested with live *S. scabiei* var. *cuniculi* mites for 48 h. Then, during the course of four-week experimental infestation period, the body weight of rabbits was recorded every two weeks for calculating body-weight variations in comparison to the non-infested control rabbits. Skin lesions in the foot area were assessed on weekly basis and serum samples were tested weekly for the estimation of changes in the total antibody levels (IgG, IgE and IgM). Moreover, DNA extracted from the blood samples was amplified for analysis of the genetic diversity in the major histocompatibility complex, class II, DQ Alpha (*MHC-DQA*) gene.

**Results:**

Compared to the IRA rabbits, the QX rabbits showed a significantly higher (*P* < 0.05) relative body weight gain compared to the non-infested control rabbits and significantly lower (*P* < 0.05) scores for foot skin lesions and higher levels of IgG, IgE and IgM at weeks 1 to 4, week 2 and week 1 post-infestation, respectively. Furthermore, a polymorphism site at position 103 bp of exon two of *MHC-DQA* gene and a different gene frequency were found between two rabbit breeds, suggesting the genetic basis for the differential host resistance to the *S. scabiei* var. *cuniculi* between two rabbit breeds.

**Conclusions:**

The QX rabbits showed higher host resistance to *S. scabiei* var. *cuniculi* compared to the IRA rabbits at the clinical, immunological and genetic levels. These results provide a reference for the breeding of rabbits with adequately improved and sustained host resistance to scabies in the domestic rabbit industry.

## Background

Scabies is a highly contagious infestation caused by *Sarcoptes scabiei*. These mites can burrow into the skin, feed on epithelial cells and serum, leading to serious skin lesions [1]. This parasitic skin disease has worldwide distribution and affects a wide range of hosts including humans, domestic animals and wildlife [2, 3]. *Sarcoptes scabiei* var. *cuniculi* has special mention as it is considered as one of the most common ectoparasites infesting rabbits. Its infestation in rabbits leads to considerable production losses (decreased productivity and weight loss), severe skin scratching and lesions and could also lead to the death in conditions of exacerbating infestation [4–6]. Therefore, bearing in mind the severe economic losses frequently occurring in rabbitries due to *S. scabiei* var. *cuniculi* infestation, it is important to develop a rabbit breed with improved host resistance to *S. scabiei* var. *cuniculi*, which will help strengthen the rabbit industry.

For testing whether the rabbit breed would be an important factor related to resistance to infestation by *S. scabiei* var. *cuniculi*, it is necessary to consider host factors at the immunological and genetic levels. Immunologically, antibody-mediated humoral immune responses are considered as the crucial immunological factors [7]. Some of the past studies have demonstrated the extremely high total IgE and IgG levels in hosts during period after infestation with *S. scabiei* [8–10] and similar responses were also observed in rabbits following the *S. scabiei* var. *cuniculi* infestation [11, 12]. Foregoing observations, to some extent, indicate that changes in the antibody levels may also serve as an adjunct reference for assessing the infestation status of *S. scabiei* var. *cuniculi* in rabbits. At the genetic level, the diversity of the major histocompatibility complex (*MHC*) is an essential reference index for monitoring the rabbit scabies. The *MHC* is closely linked by a group of highly polymorphic loci and is widespread in vertebrates, playing fundamental roles in the vertebrate immune system [13–16]. Of note, the second exon of major histocompatibility complex, class II, DQ Alpha (*MHC-DQA*) gene has been widely used in genetic diversity-related studies using the rabbit model. In particular, some studies have shown a high genetic diversity of the second exon of *MHC-DQA* gene in a comparison between rabbit breeds [17–20] and it is further believed that the genetic diversity of *MHC-DQA* gene is most likely to be driven by parasite selection [21, 22]. Therefore, the genetic diversity of the second exon of *MHC-DQA* in rabbits could also serve as a vital marker for assessing the resistance of a rabbit breed to *S. scabiei* var. *cuniculi*.

Selective breeding aimed at producing new breeds of rabbit with adequately improved and sustained host resistance to *S. scabiei* var. *cuniculi* is a viable strategy in the rabbit industry. Host resistance to parasites is an important reference indicator for selective breeding of domestic animals and it is essential to select an animal breed with high resistance to parasitic diseases for further breeding [23]. To this effect, it is necessary to consider the factors that affect breeding, such as genetic characteristics and heritability of breeding animals, conception rate, time and the environment for breeding [24].

The QiXing rabbit is a new breed of domestic rabbit propagated by the Sichuan Animal Sciences Academy, China. This breed has advantages of fast growth, high production, high feed conversion and high disease resistance. QiXing rabbits also appear to have a higher resistance to *S. scabiei* var. *cuniculi* compared to other traditional breeds in the rabbit industry, such as the IRA rabbit. However, the possible factors and putative mechanisms related to such high parasite resistance remain largely unknown.

In order to explore the possible factors having implication in *S. scabiei* var. *cuniculi* resistance between QiXing and IRA rabbit breeds, we compared the host resistance at the clinical, immunological and genetic levels. So far, there have been no reports demonstrating the comparative host resistance to *S. scabiei* var. *cuniculi* in different species and the host resistance to parasites in different rabbit breeds is also rarely reported. Therefore, the findings of our study provide a reasonable reference for adopting selective breeding strategies focusing on producing rabbit breeds with an adequately improved and sustained resistance to *S. scabiei* var. *cuniculi* in the rabbit industry.

## Methods

### Parasite and animals

The *Sarcoptes scabiei* var. *cuniculi* strain used in this study was collected from a naturally infested New Zealand White rabbit with clinical manifestations. The rabbit was obtained from a farm affected by an outbreak of scabies. Subsequently, the naturally infested rabbit was maintained along with other New Zealand White rabbits, which served as seeder rabbits.

For ensuring the desired quantity of *S. scabiei* var. *cuniculi* mites for subsequent experimental infestation, 12 New Zealand White rabbits (donor rabbits, 10 weeks-old) were infested by direct contact with the seeder rabbits for two weeks. The clinical signs in donor rabbits were evident with an estimated total body lesion area of 136 cm^2^ and about 180 live mites per cm^2^ of each donor rabbit’s affected skin in two hind limbs on average, which calculated an average of approximately 24,480 mites on two hind limbs of each donor rabbit and a total of approximately 293,760 mites on two hind limbs of all donor rabbits for further experimental infestation. For estimation of the average approximate number of *S. scabiei* var. *cuniculi* mites per cm^2^ present in the affected skin of each donor rabbit, 10 pieces of 1 cm^2^ affected skin (taken from several parts of lesions in each donor rabbit on the day of subsequent experimental infestation) were macerated in 10% KOH in test tubes and placed in boiling water until the skin was dissolved. Then the dead mites including larvae, nymphs and adults were concentrated by floating with saturated sucrose solution and the number of mites was counted using an optical microscope [25–27].

For comparative assessment of the host resistance, we used the QiXing and IRA rabbit breeds as models. The rabbits (10 weeks-old, 2–2.5 kg, 50% male) for each breed (*n* = 36 each) were generously provided by the Sichuan Animal Sciences Academy, China. All rabbits were housed individually in cages with strictly sterilized and controlled environment and provided with sterilized feed and water *ad libitum*. Furthermore, rabbits were kept under observation during the two-week acclimatization period for assuring that these rabbits were clinically and serologically free of scabies and other common infectious diseases.

### Experimental design

Two rabbit breeds were classified into two groups (*n* = 36 each), namely QX (QiXing rabbit) and IRA (IRA rabbit). In each group, 18 rabbits (half males and half females) were randomly used for setting up the experimental infestation, whereas the other 18 rabbits served as non-infested controls. The rabbits in experimentally infested QX and IRA groups were tagged as QX1–QX18 and IRA1–IRA18, respectively, whereas the rabbits in each control group were tagged as qx1–qx18 and ira1–ira18 (1–9 for males and 10–18 for females, Table 1). The initial body weight of each rabbit in the two groups was recorded and the rabbits were kept under observation before the experiment.

**Table 1:** Experimental design and allocation of rabbits in each group

Groups and numbers	Experimentally infested animals		Non-infested control	
	Male	Female	Male	Female
QiXing rabbit	QX 1–9	QX 10–18	qx 1–9	qx 10–18
IRA rabbit	IRA 1–9	IRA 10–18	ira 1–9	ira 10–18

*Note*: The control group was used to assess the body weight gain/loss in the experimentally infested rabbits only

Subsequently, for the collection of *S. scabiei* var. *cuniculi* mites, 12 donor rabbits were euthanatized on the day of experimental infestation and each experimentally infested rabbit in the QX and IRA groups was experimentally infested with *S. scabiei* var. *cuniculi* mites according to the method of Xu et al. [2], with slight modifications. Briefly, two affected limbs of each donor rabbit were shaved, burned carefully using an alcohol burner and the residues were removed using a test tube. Collected limbs of all donor rabbits were placed in Petri dishes (10 cm in diameter) at 37 °C for encouraging the mites to migrate out of the affected limbs. The mites were collected every 1 h and maintained as 0.0017–0.0020 g per sample as one inoculum containing *c.*2000 *S. scabiei* var. *cuniculi* mites (all life-cycle stages). Each inoculum was placed in a thin gauze package and then fixed on each previously shaved hind limb (foot area) of experimentally infested rabbits (*c.*4000 mites per rabbit) using an adhesive tape and left for 48 h. During this period, experimentally infested rabbits were managed carefully to ensure that no package was removed mechanically by the rabbits. It should be noted that the hind limbs were chosen to apply the mites because lesions observed in naturally infested rabbits were most frequently observed in this area.

After a 48-h period, the packages were removed from experimentally infested rabbits and infestation by *S. scabiei* var. *cuniculi* was allowed to progress further for four weeks. During the 4-week infestation period, body weight of the experimentally infested rabbits and non-infested control rabbits (serving as the controls for the assessment of body weight gain/loss of the experimentally infested rabbits only) were recorded at weeks 0, 2 and 4 post-infestation (PI). The skin lesions in the experimentally infested rabbits were assessed on a weekly basis using the index score. Blood samples were collected from marginal ear vein of the experimentally infested rabbits to separate the serum and were stored at − 20 °C for subsequent total antibody assay. In addition, blood samples collected at week 4 PI were stored at − 20 °C for extraction of DNA, amplification and genetic diversity analysis of *MHC-DQA* gene. All the experimentally infested rabbits were cured with ivermectin at the end of the infestation period.

### Clinical monitoring

The clinical parameters body weight, body weight variation, relative body weight gain and skin lesions in the experimentally infested rabbits were assessed between the QX and IRA groups. The body weight variation was assessed from the difference between body weight at week 2/4 PI and body weight at week 0 PI of the experimentally infested and non-infested control rabbits, i.e. Body weight at week 2/4 PI − Body weight at week 0 PI. The relative body weight gain was assessed from the percentage of the body weight variation relative to the initial body weight at week 0 PI of the experimentally infested and non-infested control rabbits, calculated as [(Body weight at week 2/4 PI − Body weight at week 0 PI)/ Body weight at week 0 PI] × 100%. The skin lesions were assessed by observing the inflammatory reaction in toe area of hind limb and measurements of the lesion areas were made using a caliper and the lesion scores were indexed as follows: score 0, no skin lesions; score 1, mild inflammatory reaction with reddening; score 2, severe inflammatory reaction with reddening; score 3, lesions (≤ 7.75 cm^2^) on the limbs, score 4, lesions ranging between 7.75–15.5 cm^2^ (including 15.5 cm^2^); score 5, lesions ranging between 15.5–31 cm^2^ [28, 29].

### Serum total antibody levels

Serum samples of experimentally infested rabbits in the QX and IRA groups were separated from the blood and stored at − 20 °C to test serum total antibody levels. The total IgG, IgE and IgM levels were assayed using a double-antibody sandwich enzyme-linked immunosorbent assay (ELISA) using the Rabbit IgG (Immunoglobulin G) ELISA Kit (Elabscience, Wuhan, China), Rabbit IgE (Immunoglobulin E) ELISA Kit (Elabscience) and Rabbit IgM (Immunoglobulin M) ELISA Kit (Elabscience), respectively. The procedures were performed as per the instructions of ELISA kits. Briefly, antibodies to rabbit IgG, IgM and IgE were pre-coated on ELISA plates, serum samples and calibration standard samples (used to construct a standard curve) were added to each well and detected using a detecting antibody linked to horseradish peroxidase (HRP), the color was developed with tetramethyl benzidine (TMB) after incubation and thorough washing. Color intensity was measured at 450 nm using a spectrophotometer and the OD450 nm of each sample was recorded. A standard curve of OD450 nm *versus* the concentration of circulating serum antibodies was produced to measure the concentration of circulating serum antibodies. The standard curve was generated by plotting the OD450 nm obtained from each calibration standard samples on the vertical (Y) *versus* the corresponding concentration on the horizontal (X) axis. The concentration of serum total antibodies in each serum sample was then determined by comparing the OD450 nm of the samples to the standard curve.

### Analysis of genetic diversity in *MHC-DQA* gene

Blood samples of experimentally infested rabbits in the QX and IRA groups were collected at week 4 PI and stored at − 20 °C for the extraction of genomic DNA and amplification of *MHC-DQA* gene. The blood samples were treated using an Animal Tissue Direct PCR Kit (Foregene, Chengdu, China) for extracting the genomic DNA and perform the PCR reaction. The *MHC-DQA* gene was amplified by PCR using gene-specific primers: forward (5′-TCA TCA GCT GAC CAC GTT GG-3′) and reverse (5′-GCA GCA GTA GAG TTG GAG-3′), which were designed based on the published *Oryctolagus cuniculus MHC-DQA* gene (GenBank: EU686533.1). The PCR reaction system comprised a 20-μl mix containing 10 μl of 2× PCR Easy® Mix, 7 μl of ddH_2_O, 0.5 μl of each primer (20 mM) and 2 μl of DNA template. The reaction conditions were as follows: initial denaturation at 94 °C for 3 min; followed by 30 cycles of amplification at 94 °C for 10 s, 58 °C for 20 s and 72 °C for 20 s; and a final extension step at 72 °C for 5 min. PCR products (10 μl each) were electrophoresed using a 1% agarose gel, stained with ethidium bromide and visualized on a UV transilluminator. Remaining products were used for the analysis of genetic diversity in the second exon of the *MHC-DQA* gene. PCR products used for electrophoresis were extracted and purified using a QIAquick Gel Extraction Kit (Tiangen, Beijing, China) and then cloned into vector pMD19-T (Takara, Dalian, China). Transformants were selected on Luria-Bertani agar containing ampicillin (100 mg/ml) and then subjected to automated sequencing (Invitrogen Trading Company, Shanghai, China). The DNAMAN Software (version 8.0) was used to compare the obtained nucleotide sequences of *MHC-DQA* gene with the reference sequences (GenBank: EU686533.1).

The genetic diversity on second exon of the *MHC-DQA* gene between two rabbit breeds was analyzed using the polymerase chain reaction-restriction fragment length polymorphism (PCR-RFLP) method with *Mbo* II enzyme (Takara, Beijing, China). The 15-μl reaction mix for enzyme digestion was comprised of 8 μl of PCR products, 4 μl of ddH_2_O, 2 μl of 10 × l buffer, and 1 μl of *Mbo* II enzyme. The reaction conditions for enzyme digestion were 37 °C for 8 h. For confirming the size of the restriction fragments, enzyme-digested products (10 μl each) were electrophoresed using a 3% agarose gel, stained with ethidium bromide and visualized on a UV transilluminator.

### Statistical analyses

All statistical analyses were performed using IBM SPSS statistics 20.0 (SPSS Inc., Chicago, IL, USA) software. One-way ANOVA analysis with Duncan’s multiple range test was applied to compare body weights, body weights variation, lesion score and antibody levels at different time points within the same group and at the same time point between different groups. A *P*-value < 0.05 was considered to be statistically significant. All data are presented as the mean ± standard deviation (SD). Chi-square test was performed for comparing the genotype frequency, gene frequency of the *MHC-DQA* gene in each group and a *P*-values > 0.05 was considered to be reached the Hardy–Weinberg balance. The GraphPad Prism software (version 5.01) was used for producing graphs.

## Results

### Assessment of body condition

**Fig. 1:**
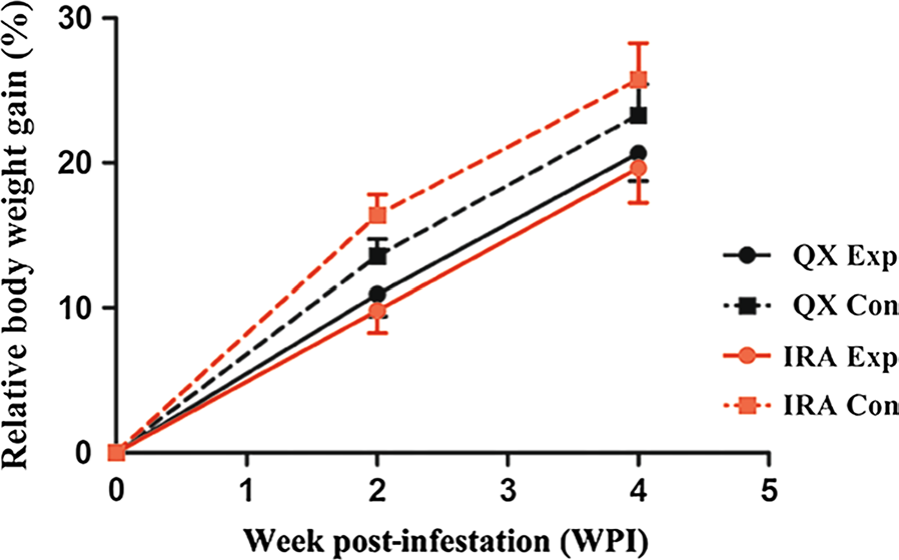
Assessment of the relative body weight gain (%) in two breeds of rabbit infested with the *S. scabiei* var. *cuniculi* and non-infested control. The body weight of each rabbit was recorded at weeks 0, 2 and 4 post-infestation (PI) and the relative body weight gain was assessed from the percentage increase in body weight of the experimentally infested and non-infested control rabbits at weeks 2 and 4 PI relative to the body weight at week 0 PI, which was calculated as [(Body weight at week 2/4 PI − Body weight at week 0 PI)/Body weight at week 0 PI] × 100%. Data points correspond to the mean of the percentage of the relative body weight gain for each group at a given time and the error bars represent the standard deviation (SD). *Abbreviations*: QX, QiXing rabbit propagated by the Sichuan Animal Sciences Academy, China; IRA, IRA rabbit breed; Exp, experimentally infested rabbits; Con, non-infested control

Before starting the experiment, initial average body weight of each breed group (*n* = 18) was calculated (Table 2). Briefly, no significant difference was observed in the initial average body weight between the experimentally infested and non-infested control rabbits within QX (*F*_(1, 34)_ = 0.031, *P* = 0.862) and IRA (*F*_(1, 34)_ = 0.048, *P* = 0.828) groups. However, the initial average body weight of both experimentally infested (*F*_(1, 34)_ = 13.163, *P* = 0.001) and non-infested control (*F*_(1, 34)_ = 13.329, *P* = 0.001) rabbits in QX group was significantly lower compared to the IRA group. During the experimental infestation period (weeks 0–4 PI), all rabbits in QX (experimentally infested: *F*_(1, 34)_ = 87.164, *P* < 0.0001; non-infested control: *F*_(1, 34)_ = 101.691, *P* < 0.0001) and IRA (experimentally infested: *F*_(1, 34)_ = 57.117, *P* < 0.0001; non-infested control: *F*_(1, 34)_ = 108.363, *P* < 0.0001) groups showed a significant increase in average body weight. Meanwhile, body weight variation (weeks 0–2 PI: *F*_(3, 68)_ = 150.413, *P* < 0.0001; weeks 0–4 PI: *F*_(3, 68)_ = 161.605, *P* < 0.0001) and the relative body weight gain (weeks 0–2 PI: *F*_(3, 68)_ = 81.782, *P* < 0.0001; weeks 0–4 PI: *F*_(3, 68)_ = 26.530, *P* < 0.0001) in the non-infested control rabbits were significantly higher compared to the experimentally infested rabbits in both groups (Fig. 1, Table 2). The body weight variation (weeks 0–2 PI: *F*_(1, 34)_ = 3.567, *P* = 0.067; weeks 0–4 PI: *F*_(1, 34)_ = 3.970, *P* = 0.082) and relative body weight gain (weeks 0–2 PI: *F*_(1, 34)_ = 0.403, *P* = 0.501; weeks 0–4 PI: *F*_(1, 34)_ = 2.060, *P* = 0.160) in the experimentally infested QX rabbits showed no significant difference compared to the IRA rabbits; however, values for body weight variation (weeks 0–2 PI: *F*_(1, 34)_ = 107.071, *P* < 0.0001; weeks 0–4 PI: *F*_(1, 34)_ = 163.155, *P* < 0.0001) and relative body weight gain (weeks 0–2 PI: *F*_(1, 34)_ = 42.896, *P* < 0.0001; weeks 0–4 PI: *F*_(1, 34)_ = 9.864, *P* = 0.003) were significantly lower in the QX non-infested control rabbits compared to the IRA (Fig. 1, Table 2), suggesting that difference in the relative body weight gain with respect to the corresponding control is higher in the experimentally infested IRA rabbits. Hence, the experimentally infested IRA rabbits gained less weight compared to the QX rabbits over the course of the experiment, indicating that the IRA rabbits were relatively less resistant and hence affected more following infestation. The line chart in Fig. 1 depicts the results intuitively as the growth trend of the relative body weight gain between the experimentally infested and non-infested control rabbits was closer in QX group compared to the IRA rabbits.

**Table 2:** Average body weight, body weight variation and relative body weight gain in two rabbit breeds experimentally infested with *S. scabiei* var. *cuniculi* and non-infested control

WPI	Body weight (g) (mean ± SD)				Body weight variation (g) (mean ± SD)				Relative body weight gain (%) (mean ± SD)			
	QX		IRA		QX		IRA		QX		IRA	
	Exp	Con	Exp	Con	Exp	Con	Exp	Con	Exp	Con	Exp	Con
0	2280.44 ± 153.51^b(c)^	2271.33 ± 158.68^b(c)^	2495.33 ± 198.95^a(c)^	2481.33 ± 185.41^a(c)^	0.00 ± 0.00^a(c)^	0.00 ± 0.00^a(c)^	0.00 ± 0.00^a(c)^	0.00 ± 0.00^a(c)^	0.00 ± 0.00^a(c)^	0.00 ± 0.00^a(c)^	0.00 ± 0.00^a(c)^	0.00 ± 0.00^a(c)^
2	2528.28 ± 144.60^c(b)^	2578.72 ± 170.90^bc(b)^	2737.78 ± 191.25^ab(b)^	2886.67 ± 196.75^a(b)^	247.83 ± 24.35^c(b)^	307.39 ± 26.92^b(b)^	242.44 ± 23.14^c(b)^	405.33 ± 29.80^a(b)^	10.94 ± 1.52^c(b)^	13.56 ± 1.17^b(b)^	9.81 ± 1.54^c (b)^	16.40 ± 1.41^a (b)^
4	2749.28 ± 147.74^c(a)^	2797.44 ± 154.32^bc(a)^	2980.94 ± 186.38^ab(a)^	3116.11 ± 180.44^a(a)^	468.83 ± 21.12^c(a)^	526.11 ± 24.60^b(a)^	485.61 ± 27.04^c(a)^	634.78 ± 26.41^a(a)^	20.67 ± 1.93^c(a)^	23.29 ± 2.15^b(a)^	19.63 ± 2.39^c (a)^	25.74 ± 2.53^a (a)^

*Note*: Body weight variation represents the difference between the body weight at week 2/4 PI and the body weight at week 0 PI; relative body weight gain represents the percentage of the body weight variation relative to the initial body weight at week 0 PI. The different superscript letters within a row denote significant differences between different groups (*P* < 0.05). The different superscript letters in parentheses within a column denote significant differences between different time points (*P* < 0.05)

*Abbreviation*: QX, QiXing rabbit breed propagated by the Sichuan Animal Science Academy, China; IRA, IRA rabbit breed; WPI, week post-infestation; Exp, experimentally infested animals; Con, non-infested control; SD, standard deviation

### Assessment of clinical manifestation and skin lesions

**Fig. 2:**
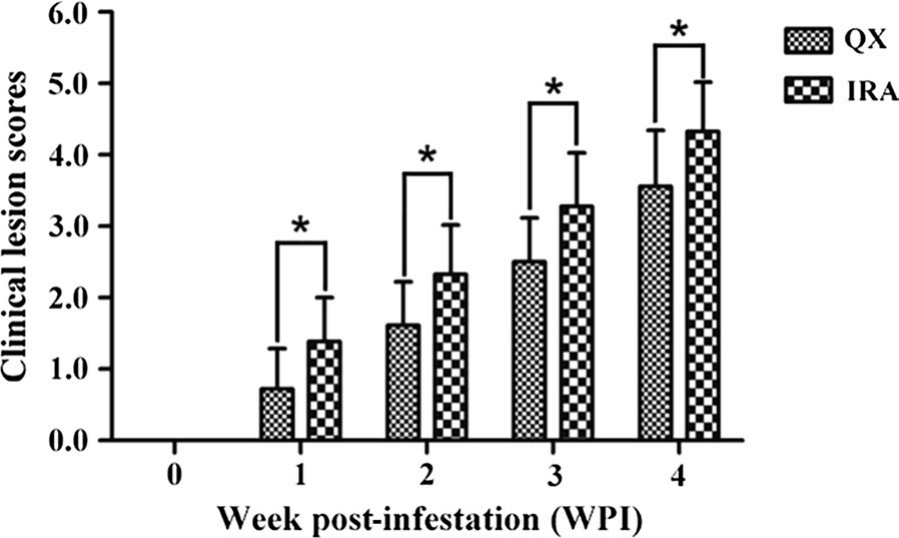
Differential assessment of the skin lesions at each week post-infestation in two rabbit breeds infested with *S. scabiei* var. *cuniculi.* The assessment was made using the lesion score method. The lesion scores in the experimentally infested rabbits were recorded on weekly basis. Skin lesions were scored on a 0–5 grade based on the inflammatory reaction in toe area of the hind limb as well as the lesion areas of the experimentally infested rabbits. Histogram represents the mean of the lesion scores for each group at a given time and the error bars represent the standard deviation (SD). Asterisks indicate statistically significant differences in the lesion score between two different groups of rabbits (*P < 0.05). *Abbreviations*: QX, the QiXing rabbit propagated by the Sichuan Animal Sciences Academy, China; IRA, the IRA rabbit

During the infestation period (weeks 0–4 PI), all the experimentally infested rabbits in the QX (*F*_(1, 34)_ = 153.000, *P* < 0.0001) and IRA (*F*_(1, 34)_ = 185.809, *P* < 0.0001) groups developed progressive skin lesions caused by infestation of *S. scabiei* var. *cuniculi*, showing a significant increase in the average lesion score over the course of time (Fig. 2, Table 3). Clinical manifestations in the experimentally infested rabbits in two groups first became visible at week 1 PI, appearing as mild inflammation in toe area of hind limb and itching symptoms in rabbits. As the infestation period increased, the experimentally infested rabbits showed more severe clinical signs in two groups, with reddening and significant inflammation in toe area of hind limb skin and apparent itching symptoms in rabbits at week 2 PI. The skin lesions on the hind limb of the experimentally infested rabbits were first observed in both groups at week 3 PI and then became more apparent during the period between weeks 3 and 4 PI. At week 4 PI, all experimentally infested rabbits in both groups showed apparent scabbing with the typical clinical signs of *S. scabiei* var. *cuniculi* infestation.

**Table 3:** Average lesion score in the two breeds of rabbits experimentally infested with *S. scabiei* var. *cuniculi*

WPI	Lesion scores (points) (mean ± SD)	
	QX	IRA
0	0.00 ± 0.00^a(e)^	0.00 ± 0.00^a(e)^
1	0.72 ± 0.57^b(d)^	1.39 ± 0.61^a(d)^
2	1.61 ± 0.61^b(c)^	2.33 ± 0.69^a(c)^
3	2.50 ± 0.62^b(b)^	3.28 ± 0.75^a(b)^
4	3.56 ± 0.78^b(a)^	4.33 ± 0.69^a(a)^

*Note*: Skin lesions were scored on a 0–5 grade based on the inflammatory reaction in toe area of the hind limb as well as the lesion areas of the experimentally infested rabbits. The different superscript letters within a row denote significant differences between different groups (*P* < 0.05). The different superscript letters in parentheses within a column denote significant differences between the different time points (*P* < 0.05)

*Abbreviation*: QX, QiXing rabbit breed propagated by the Sichuan Animal Science Academy, China; IRA, IRA rabbit breed; WPI, week post-infestation; SD, standard deviation

The average lesion score (mean ± SD) of experimentally infested rabbits in the two groups are shown in Table 3. During the infestation period (weeks 0 to 4 PI), the experimentally infested rabbits in QX group showed a significantly lower lesion score compared to the IRA group at weeks 1 to 4 PI (week 1 PI: *F*_(1, 34)_ = 11.439, *P* = 0.002; week 2 PI: *F*_(1, 34)_ = 11.179, *P* = 0.002; week 3 PI: *F*_(1, 34)_ = 11.490, *P* = 0.002; week 4 PI: *F*_(1, 34)_ = 10.036, *P* = 0.003), together with less serious clinical signs compared to the IRA group. Meanwhile, the histogram in Fig. 2 intuitively depicts the significantly higher lesion score of IRA compared to the QX. These results indicated that the IRA rabbits exhibited more serious clinical signs of infestation of *S. scabiei* var. *cuniculi* compared to the QX rabbits.

### Serum antibody responses

**Fig. 3:**
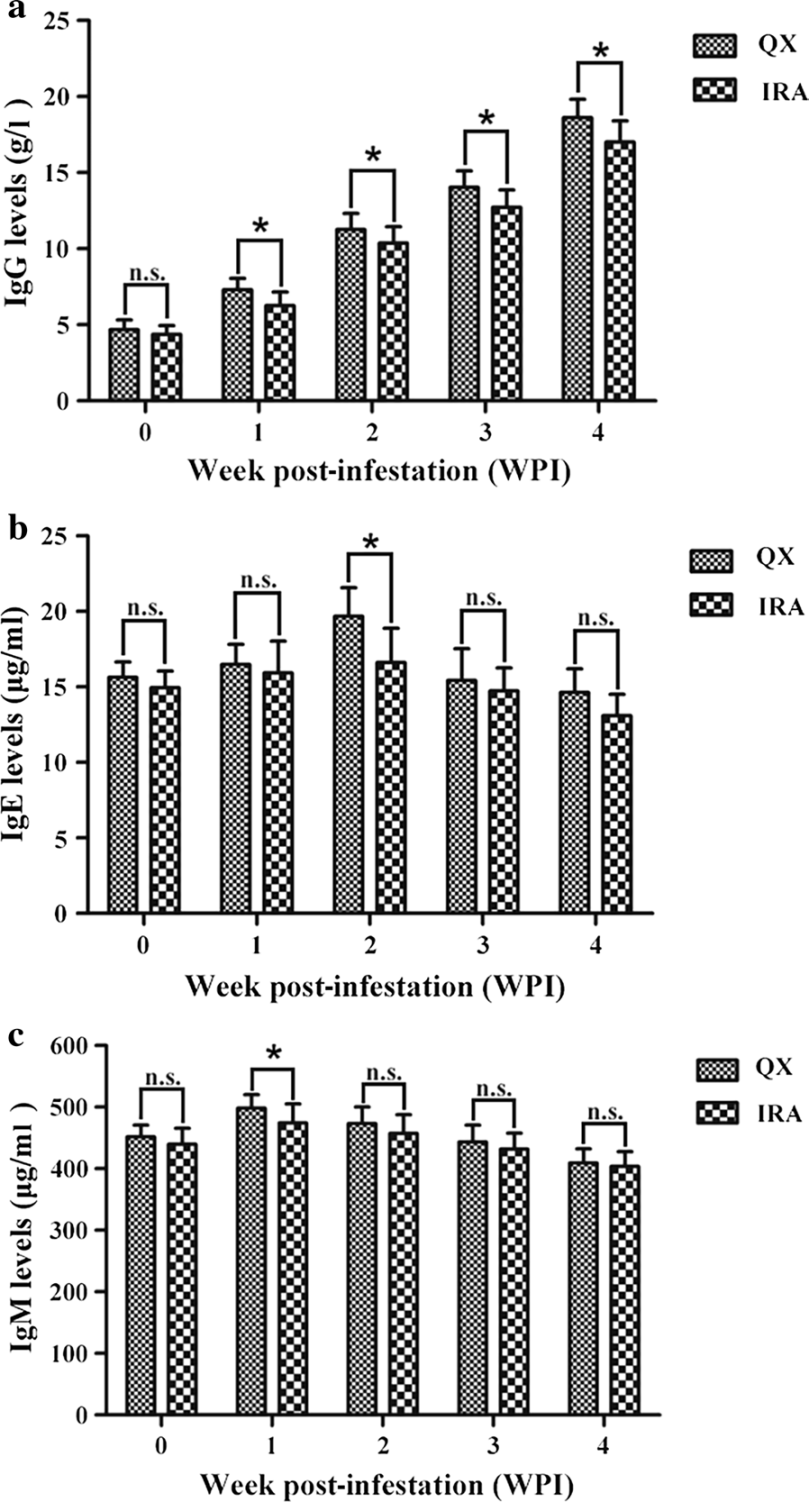
Differential assessment of serum total antibody levels at each week post-infestation in two rabbit breeds infested with *S. scabiei* var. *cuniculi.* The serum samples were collected from the experimentally infested rabbits on weekly basis for assaying the total antibody levels by an enzyme-linked immunosorbent assay (ELISA) using the commercial kits. **a** Average total IgG levels in experimentally infested rabbits in two breeds at each week post-infestation (g/l). **b** Average total IgE levels in experimentally infested rabbits in two breeds at each week post-infestation (μg/ml). **c** Average total IgM levels in experimentally infested rabbits in two breeds at each week post-infestation (μg/ml). The histogram represents the mean of the total antibody levels for each group at a given time and the error bars represent the standard deviation (SD). Asterisks indicate statistically significant differences in the total antibody levels between two different groups of rabbit (*P < 0.05) and ‘n.s.’ indicates non-significant differences. *Abbreviations*: QX, the QiXing rabbit propagated by the Sichuan Animal Sciences Academy, China; IRA, the IRA rabbit

During the infestation period (weeks 0–4 PI), the antibody-mediated humoral immune responses in the two groups of experimentally infested rabbits were elicited robustly. Analysis of the initial average total antibody levels at week 0 PI showed no significant difference in the experimentally infested rabbits of the two groups (IgG: *F*_(1, 34)_ = 2.355, *P* = 0.134; IgE: *F*_(1, 34)_ = 3.653, *P* = 0.064; IgM: *F*_(1, 34)_ = 2.535, *P* = 0.121). The variation in serum total antibody levels became more apparent as the infestation progressed and the comparison of antibody levels at each week PI showed differences between the experimentally infested rabbits in the two breed groups (Fig. 3, Table 4).

**Table 4:** Average total antibody levels (IgG, IgE and IgM) in the two rabbit breeds experimentally infested with *S. scabiei* var. *cuniculi*

WPI	IgG levels (g/l) (mean ± SD)		IgE levels (μg/ml) (mean ± SD)		IgM levels (μg/ml) (mean ± SD)	
	QX	IRA	QX	IRA	QX	IRA
0	4.68 ± 0.65^a(e)^	4.36 ± 0.58^a(e)^	15.63 ± 1.02^a(b)^	14.95 ± 1.11^a(bc)^	451.79 ± 18.99^a(c)^	439.72 ± 25.96^a(b)^
1	7.30 ± 0.76^a(d)^	6.26 ± 0.90^b(d)^	16.49 ± 1.33^a(b)^	15.92 ± 2.12^a(ab)^	498.35 ± 22.08^a(a)^	474.02 ± 31.01^b(a)^
2	11.26 ± 1.07^a(c)^	10.36 ± 1.08^b(c)^	19.68 ± 1.89^a(a)^	16.62 ± 2.27^b(a)^	473.13 ± 26.89^a(b)^	457.24 ± 30.50^a(ab)^
3	14.03 ± 1.09^a(b)^	12.71 ± 1.16^b(b)^	15.42 ± 2.12^a(b)^	14.73 ± 1.53^a(bc)^	443.14 ± 27.47^a(c)^	431.89 ± 26.02^a(b)^
4	18.61 ± 1.21^a(a)^	17.02 ± 1.38^b(a)^	14.63 ± 1.57^a(b)^	13.10 ± 1.41^a(c)^	409.15 ± 22.85^a(d)^	403.68 ± 24.08^a(c)^

*Note*: Total antibody levels were measured by an enzyme-linked immunosorbent assay (ELISA) using commercial kits. The different superscript letters within a row denote significant differences between different groups (*P* < 0.05). The different superscript letters in parentheses within a column denote significant differences between the different time points (*P* < 0.05)

*Abbreviations*: QX, QiXing rabbit breed propagated by the Sichuan Animal Science Academy, China; IRA, IRA rabbit breed; WPI, week post-infestation; SD, standard deviation

After experimental infestation with *S. scabiei* var. *cuniculi*, the rabbits developed a gradual IgG response. Both groups of experimentally infested rabbits showed a progressive increase in IgG levels from weeks 0–4 PI (Fig. 3a) and the IgG levels in experimentally infested rabbits in the QX group were significantly higher compared to those in the IRA group during weeks 1–4 PI (week 1 PI: *F*_(1, 34)_ = 14.055, *P* = 0.001; week 2 PI: *F*_(1, 34)_ = 6.324, *P* = 0.017; week 3 PI: *F*_(1, 34)_ = 12.299, *P* = 0.001; week 4 PI: *F*_(1, 34)_ = 13.487, *P* = 0.001) (Fig. 3a, Table 4).

Similarly, the IgE responses to infestation with *S. scabiei* var. *cuniculi* also demonstrated a variation in the IgE levels in the experimentally infested rabbits in the two groups. The IgE levels in the experimentally infested rabbits in both groups increased from weeks 0–2 PI, and then progressively decreased to the initial level from weeks 2–4 PI. As depicted in Fig. 3b and Table 4, there was no significant difference in IgE levels between the experimentally infested rabbits in the two breed groups from weeks 0 to week 1 PI (*F*_(1, 34)_ = 0.935, *P* = 0.340). However, at week 2 PI, the IgE levels in the QX experimentally infested rabbits were significantly higher compared to those in the IRA rabbits (*F*_(1, 34)_ = 19.307, *P* < 0.0001). From weeks 3–4 PI, the differences between the two groups of experimentally infested rabbits decreased and there were only slightly, but not significantly, higher IgE levels in the QX experimentally infested rabbits compared to the IRA rabbits (week 3 PI: *F*_(1, 34)_ = 1.243, *P* = 0.273; week 4 PI: *F*_(1, 34)_ = 2.218, *P* = 0.146) (Fig. 3b, Table 4).

IgM is the first antibody raised in response to antigen exposure and its response to *S. scabiei* var. *cuniculi* was crucial to show the differences of antibody-mediated humoral immune responses of experimentally infested rabbits between two breed groups. Intriguingly, the IgM levels of the experimentally infested rabbits in both breed groups increased from weeks 0–1 PI and then progressively decreased to the initial level from weeks 1–3 PI and further lowered below the initial level at week 4 PI. As shown in Fig. 3c and Table 4, the IgM levels in the experimentally infested rabbits in QX group were significantly higher compared to those in the IRA group at week 1 PI (*F*_(1, 34)_ = 7.348, *P* = 0.010). However, for the rest of the infestation period there were only slightly but not significantly, higher IgM levels were observed in the QX experimentally infested rabbits compared to the IRA rabbits (week 2 PI: *F*_(1, 34)_ = 2.746, *P* = 0.107; week 3 PI: *F*_(1, 34)_ = 1.592, *P* = 0.216; week 4 PI: *F*_(1, 34)_ = 0.489, *P* = 0.489) (Fig. 3c, Table 4).

### Amplification and genetic diversity analysis of *MHC-DQA* gene

**Fig. 4:**
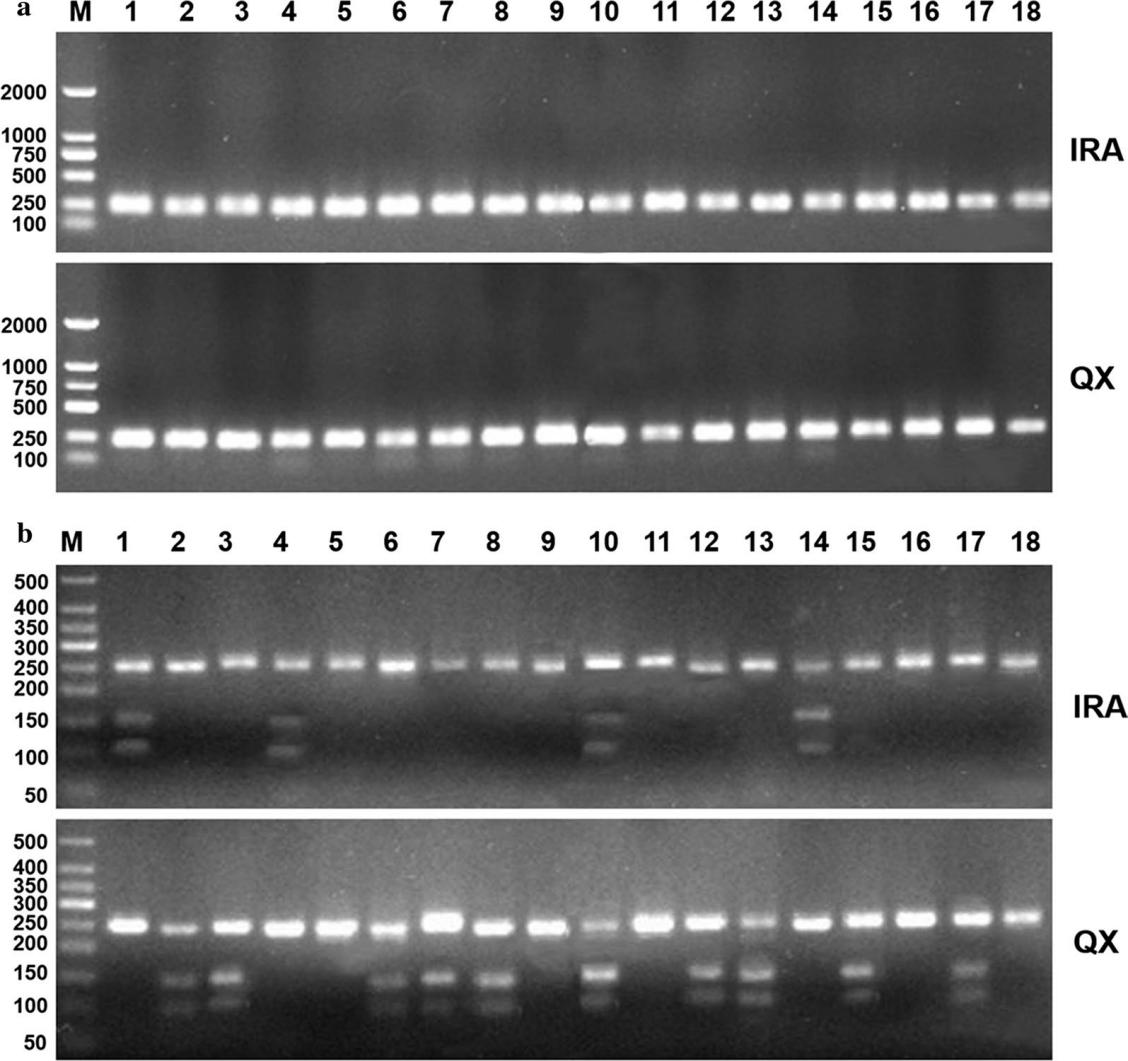
Agarose gel electrophoresis for the amplification and genetic diversity analysis of *MHC-DQA* gene. The blood samples were collected from the experimentally infested rabbits at week 4 post-infestation for amplification of MHC class II DQA gene by direct PCR, and the genetic diversity on the second exon of *MHC-DQA* gene between two breeds of rabbit were analyzed by the polymerase chain reaction-restriction fragment length polymorphism (PCR-RFLP) method with *Mbo* II enzyme. **a** Amplification of *MHC-DQA* gene in QiXing rabbits and IRA groups. Lane M: DL2000 DNA marker; Lanes 1–18: amplicons from experimentally infested rabbits in each group. **b** Analysis of genetic diversity in *MHC-DQA* gene by PCR-RFLP method with *Mbo* II enzyme. Lane M: D50 DNA marker; Lanes 1–18: amplicons from the experimentally infested rabbits in each group. *Abbreviations*: QX, the QiXing rabbit propagated by the Sichuan Animal Sciences Academy, China; IRA, the IRA rabbit

The *MHC-DQA* gene was successfully amplified from genomic DNA extracted from blood samples of experimentally infested rabbits in the QX and IRA groups using gene-specific primers. After electrophoresis through an agarose gel, PCR amplification products showed a single clear band of approximately 250 bp, which corresponded to the expected size of the PCR products (248 bp) of the target gene fragment (Fig. 4a). All PCR amplification products used for electrophoresis in two groups were extracted and purified and then successfully cloned into the vector pMD19-T for sequencing. The sequenced results of *MHC-DQA* gene were then compared with the published *Oryctolagus cuniculus MHC-DQA* gene in GenBank, which showed an average identity of 99.5%. Besides, one cutting site for *Mbo* II restriction enzyme was identified at position “103” of *MHC-DQA* gene sequence.

After *Mbo* II digestion reaction of the PCR products, two single clear bands with sizes of near 145 bp and 103 bp were observed after electrophoresis through an agarose gel in the partial PCR products from the QX and IRA breed groups (Fig. 4b), which confirmed that genetic diversity at position 103 bp of the second exon of *MHC-DQA* gene existed between the two rabbit breeds. In addition, there was a corresponding difference in the gene frequency between the QX and IRA groups. It could be inferred from Fig. 4b that there were two alleles of the second exon of *MHC-DQA* gene, which were named as allele A (without the restriction enzyme cutting site for *Mbo* II at 103 bp) and allele B (with the restriction enzyme cutting site for *Mbo* II at 103 bp). Besides, two genotypes were derived from two alleles, which were named as AA (without the restriction enzyme cutting site and only a 248-bp band was observed) and AB (with the restriction enzyme cutting site and 248 bp, 103 bp and 145 bp bands were observed). From the PCR products in the QX and IRA groups, 10 out of 18 PCR products amplified from serum samples collected from experimentally infested rabbits in the QX group had the restriction enzyme cutting site for *Mbo* II at 103 bp; however, only 4 out of 18 PCR products in the IRA group had the restriction enzyme cutting site (Fig. 4b). Hence, according to the alleles of the second exon of *MHC-DQA* gene (allele A and B), the genotypes of *MHC-DQA* gene were 8 AA and 10 AB in QX and 14 AA and 4 AB in IRA (Table 5).

**Table 5:** Allele and genotype frequencies of the *MHC* class II DQA gene exon two in the two rabbit breeds experimetally infested with *S. scabiei* var. *cuniculi*

Group	Genotype frequency		Allele frequency		*χ*^2^
	AA	AB	A	B	
QX	0.444 (*n* = 8)	0.556 (*n* = 10)	0.772	0.278	2.663
IRA	0.778 (*n* = 14)	0.222 (*n* = 4)	0.889	0.111	0.281

*Note*: The genetic diversity of the MHC class II DQA gene exon two was analyzed by the polymerase chain reaction-restriction fragment length polymorphism (PCR-RFLP) method with *Mbo* II enzyme. χ^2^ test showed that the restriction enzyme cutting site for *Mbo* II reached the Hardy–Weinberg equilibrium in two breed groups (*P* > 0.05)

*Abbreviations*: QX, QiXing rabbit breed propagated by the Sichuan Animal Science Academy, China; IRA, IRA rabbit breed; A, allele A; B, allele B

In terms of the second exon of *MHC-DQA* gene, the AB genotype was dominant in the experimentally infested rabbits of the QX group, with a genotype frequency of 0.556 and the AA genotype was dominant in the IRA experimentally infested rabbits, with a genotype frequency of 0.778. For the alleles of the second exon of *MHC-DQA* gene, allele A was the dominant allele both in the experimentally infested QX rabbits (0.772 gene frequency) and experimentally infested IRA rabbits (0.889 gene frequency) (Table 5). The Chi-square test showed that the restriction enzyme cutting site for *Mbo* II in *MHC-DQA* gene reached the Hardy–Weinberg equilibrium in the experimentally infested rabbits of QX (*χ*^2^ = 2.663, *df* = 1, *P* = 0.103) and IRA (*χ*^2^ = 0.281, *df* = 1, *P* = 0.596) groups.

For further investigating the differences in the clinical condition of genotype AA and AB individuals in the two breeds, the average relative body weight gain and lesion scores at week 4 PI were compared between genotype AA and AB individuals within each breed group and between the two breed groups (Table 6). Results showed that the AB individuals showed significantly higher average relative body weight gain (QX: *F*_(1, 16)_ = 33.966, *P* < 0.0001; IRA: *F*_(1, 16)_ = 15.279, *P* = 0.001) and significantly lower average lesion scores (QX: *F*_(1, 16)_ = 12.937, *P* = 0.002; IRA: *F*_(1, 16)_ = 12.903, *P* = 0.002) than AA individuals in the two breed groups. Meanwhile, both the AA and AB individuals between the two breed groups showed no significant difference in average relative body weight gain (QX: *F*_(1, 20)_ = 0.070, *P* = 0.793; IRA: *F*_(1, 12)_ = 0.499, *P* = 0.494) and lesion scores (QX: *F*_(1, 20)_ = 3.219, *P* = 0.088; IRA: *F*_(1, 12)_ = 1.407, *P* = 0.259).

**Table 6:** Average relative body weight gain and lesion scores at week 4 post-infestation in AA and AB individuals between the two rabbit breeds experimentally infested with *S. scabiei* var. *cuniculi*

Group	Genotype	Relative body weight gain (%) (mean ± SD)	Lesion scores (points) (mean ± SD)
QX	AA (*n* = 8)	18.15 ± 1.27^a^	3.84 ± 0.76^a^
	AB (*n* = 10)	23.11 ± 1.49^b^	2.98 ± 0.83^b^
IRA	AA (*n* = 14)	17.84 ± 2.06^a^	4.06 ± 0.63^a^
	AB (*n* = 4)	22.94 ± 1.85^b^	3.65 ± 0.54^a^

*Note*: The data were collected in week 4 post-infestation. The different superscript letters within a column denote significant differences between groups (*P* < 0.05)

*Abbreviations*: QX, QiXing rabbit breed propagated by the Sichuan Animal Science Academy, China; IRA, IRA rabbit breed; SD, standard deviation

## Discussion

The difference in host resistance to infestation with parasites among different breeds of host animals is an attractive topic for investigation. Previous studies focusing on the host resistance in different breeds of domestic animals have mainly centered on cattle and sheep as models and involving the parasites (largely ectoparasites) such as *Boophilus microplus*, *Amblyomma hebraeum*, *Psoroptes ovis*, and *Ostertagia circumcincta* [30–34]. The most attractive study on this topic was that performed by Charlotte et al. [34], who investigated the comparative differences in cutaneous and *in vitro* cellular immune responses between two different cattle breeds (Belgian Blue beef and Holstein-Friesians) during a natural infestation with *Psoroptes ovis* and revealed differences in the host resistance between the two cattle breeds at the immunological level. In the present study, we focused on one of the most common parasites infesting rabbits, i.e. *S. scabiei* var. *cuniculi*, and comprehensively investigated the differential host resistance to this parasite in two different rabbit breeds at the clinical, immunological and genetic levels.

It has been demonstrated that infestation with *S. scabiei* var. *cuniculi* in rabbits causes serious scratching with skin lesions and considerable weight loss [4] and the clinical condition represented by relative body weight gain and skin lesions can intuitively reflect the severity of the infestation in rabbits between different breeds. The relative body weight gain and skin lesions have been regarded as the reference markers for comparing the influence on the immunological responses to *S. scabiei* var. *cuniculi* between different methods of infestation (by contact or by means of a dressing), it has been shown that these two clinical indices are important in evaluating the severity of the *S. scabiei* var. *cuniculi* infestation in rabbits [28]. Intriguingly, when compared to the QiXing rabbits, our results also showed that IRA rabbits gained less weight and a significantly higher lesion score (week 1 PI: *P* = 0.002; week 2 PI: *P* = 0.002; week 3 PI: *P* = 0.002; week 4 PI: *P* = 0.003) together with more serious clinical signs during the infestation with *S. scabiei* var. *cuniculi*. These differences in clinical condition between the two rabbit breeds provide evidence that the QiXing breed displayed better clinical signs compared to the IRA breed following infestation with *S. scabiei* var. *cuniculi* and preliminarily indicated that differences in the host resistance to *S. scabiei* var. *cuniculi* existed between these two breeds of rabbit.

The gradual IgG response developed in both rabbit breeds with a progressive increase in the IgG levels. These findings corroborate similar trends reported in previous studies focusing on antibody responses to *S. scabiei* var. *cuniculi* in rabbits [11, 12]. Meanwhile, studies in dogs and goats also showed similar trends following primary infestation with *S. scabiei* [35–37], indicating that the progressive increase in the total IgG levels in the two breeds was associated with the infestation. Our results also showed that the total IgG levels in QX rabbits were significantly higher compared to the IRA rabbits during the infestation period (week 1 PI: *P* = 0.001; week 2 PI: *P* = 0.017; week 3 PI: *P* = 0.001; week 4 PI: *P* = 0.001), indicating that the QX rabbits developed a stronger IgG response to *S. scabiei* var. *cuniculi* infestation compared to the IRA breed.

IgE is an important component of the host defense against a variety of parasites and along with mast cells, basophils and eosinophils, serves as an essential element in allergic and parasitic inflammation [7]. Similar to the levels of IgG, IgE were also found to be elevated in previous studies on rabbits, sheep and goats [37–39]. In our study, the IgE levels between the two rabbit breeds were only significantly different at week 2 PI, with QX rabbits showing significantly higher levels than IRA (*P* < 0.0001), indicating that the QX rabbits developed a stronger IgE response to the infestation compared to the IRA rabbits.

IgM was the first antibody to appear in response to the antigen exposure from *S. scabiei* var. *cuniculi* and our results showed that the IgM levels in the two rabbit breeds during infestation were increased during the week 1 PI and significantly differed between the two groups only at week 1 PI (*P* = 0.010), but progressively decreased below the initial levels during the remaining infestation period. This was in agreement with the variation trend of the IgM response to infestation of *S. scabiei* reported previously [11], indicating that the IgM response was elicited by the infestation and a specific IgM immune response was developed. Additionally, significantly higher IgM levels in QX rabbits compared to the IRA rabbits at week 1 PI indicated a higher IgM response to *S. scabiei* var. *cuniculi* infestation developed in QX rabbits (*P* = 0.010). Hence, the differences in IgG, IgE and IgM responses between these two rabbit breeds demonstrate that the host resistance to *S. scabiei* var. *cuniculi* differed between them being comparatively higher in the QX breed.

High polymorphism and multi-base mutations are the prominent features in *MHC* gene in vertebrates and closely associated with differential host resistance to parasitic infestation [40, 41]. To date, 19 rabbit *MHC-DQA* alleles have been reported showing high levels of polymorphism at the rabbit DQA locus [17]. In rabbits, Oppelt et al. [42] evaluated the potential associations between individual *MHC* class II DRB constitutions and the rabbits’ intestinal burden of nematodes and coccidia and demonstrated that rabbits with a particular allele of *MHC* gene showed a lower level of infestation with *Eimeria stiedai*. In our study, two alleles (A and B) for the second exon of *MHC-DQA* gene were identified using PCR-RFLP with *Mbo* II. Allele A was the dominant in both the QX rabbits (0.772 gene frequency) and the IRA rabbits (0.889 gene frequency). However, in terms of genotype frequency, the AB genotype was dominant in the QX breed (0.556 genotype frequency) and the AA genotype was dominant in the IRA breed (0.778 genotype frequency). Combined with the cloned and sequenced results of *MHC-DQA* gene PCR amplification products, all the AB genotype clones were identical in both QX and IRA breed individuals. Meanwhile, the comparation of average relative body weight gain and lesion scores between AA and AB individuals at week 4 PI within each breed group and between the two breed groups, showed that AB individuals had a significantly higher average relative body weight gain (QX: *P* < 0.0001; IRA: *P* = 0.001) and significantly lower average lesion scores (QX: *P* = 0.002; IRA: *P* = 0.002) than AA individuals in the two breed groups. These findings indicate that the differences in host resistance to *S. scabiei* var. *cuniculi* between the two rabbit breeds were likely under the regulation and control of *MHC-DQA* allele B. In addition to the differences in host resistance to *S. scabiei* var. *cuniculi* at the clinical and immunological levels as discussed above, QX rabbits with the advantageous AB genotype (heterozygous with two alleles) showed a higher resistance to *S. scabiei* var. *cuniculi* infestation compared to the IRA rabbits. These results also corroborate the findings of Oppelt et al. [42], who showed that heterozygous genotype with two alleles had a lower parasite load and a higher resistance to the infestation.

The existence of genetic diversity in *MHC-DQA* gene and appearance of the alleles are believed to be driven by selection for resistance to parasites and other pathogens, which are themselves driven to evolve the mechanisms for escaping the host immune responses [17]. Such selection often involves balancing selection, which operates by an overdominance or frequency dependence and can maintain the polymorphic alleles for considerable periods of time [43]. The IRA rabbit is a traditional breed in the domestic rabbit industry and the QiXing rabbit was developed by hybridizing two useful breeds. The maternal line of this new breed (QiXing) is an advantage breed of rabbit from Sichuan maintained by long-term evolution and adaptation, with excellent resistance to parasites and other pathogens. Hence, the differences in genotype and gene frequency on the exon two of *MHC-DQA* gene in the host are likely because the polymorphism of each allele was driven by a long-term selection for the resistance to parasites and other pathogens and the differences in the host resistance of rabbits to the infestation of parasites are directly related to the genetic diversity of exon two of *MHC-DQA* gene.

In summary, using clinical condition assessment, humoral immune responses and analysis of the genetic diversity of the *MHC-DQA* gene, we observed sufficient differences in host resistance between two rabbit breeds following an experimental infestation with *S. scabiei* var. *cuniculi*. The findings of our study provide foundation for further research focusing on elucidation of the potential mechanism implicated in modulating the *MHC-DQA* alleles associated with *S. scabiei* var. *cuniculi* infestation. Further studies aimed at identifying other potential factors implicated in the differential host resistance to the *S. scabiei* var. *cuniculi* between these two rabbit breeds would be of a great value for understating the basis of differential breed susceptibility to *S. scabiei* var. *cuniculi*.

## Conclusions

In this study we compared the host resistance to *S. scabiei* var. *cuniculi* between the QiXing and IRA rabbit breeds at the clinical, immunological and genetic levels, and revealed a differential host resistance to *S. scabiei* var. *cuniculi* between the two rabbit breeds. Intriguingly, the QiXing rabbit breed showed a relatively higher resistance to *S. scabiei* var. *cuniculi* infestation compared to the IRA rabbit breed. These results not only provide a reference for breeding of rabbits with adequately improved resistance to *S. scabiei* var. *cuniculi* in the domestic rabbit industry, but also provide reasonable foundation for further studies focusing on the differences in parasite resistance in other host species.

## Data Availability

The datasets supporting the conclusions of this article are included within the article.

## Abbreviations

QX: QiXing rabbit
IRA: IRA rabbit
*MHC-DQA*: major histocompatibility complex, class II, DQ alpha gene
PI: post-infestation
HRP: horseradish peroxidase
TMB: tetramethyl benzidine
ELISA: enzyme-linked immunosorbent assay
PCR: polymerase chain reaction
PCR-RFLP: polymerase chain reaction-restriction fragment length polymorphism
